# Evolution Mechanism of Photonically Sintered Nano-Silver Conductive Patterns

**DOI:** 10.3390/nano9020258

**Published:** 2019-02-14

**Authors:** Fanbo Meng, Jin Huang

**Affiliations:** Department of Mechano-Electronic Engineering, School of Xidian University, Xi’an 710000, China; fbmeng0412@163.com

**Keywords:** conductivity, flash sintering, inkjet printing, sintering neck

## Abstract

Flash sintering is the most promising sintering method because of its high speed and large area of effect. However, current flash sintering processes exhibit poor stability and the conductive pattern surface is highly susceptible to damage during this process. Therefore, a sintering parameter prediction system must be established to optimize sintering parameters for manufacturing. In this study, a photon-sintered nano-silver particle model is proposed for studying the sintering characteristics of metal nanoparticles. The temperature field of the sintering area is obtained using the heat transfer formula and the sintered neck state, and the conductive pattern density of the nano-silver particles are obtained by employing the fluid dynamics finite element method. The conductive pattern’s structural density and conductivity are determined using the electronic state density and potential distribution of the crystal structure. The sintering state is then predicted based on the sintering parameters. The simulation results are consistent with conductive patterns corresponding to different sintering degrees observed using an electron microscope. The results of this study provide reference sintering parameters for flash sintering with effective cost reduction.

## 1. Introduction

The use of inkjet printing technology [1] to produce conductive patterns [2] is more efficient and less expensive than traditional methods such as photolithography and etching. When using inkjet printing to create a conductive pattern, the conductive pattern must be sintered to ensure electrical conductivity and adherence to the substrate. At present, the method used for sintering is oven heating [3,4]; the conductive pattern must be placed in a high-temperature environment for a long period to improve electrical conductivity. For substrates that are easily damaged or that have strict requirements, the high temperatures required can cause problems such as low efficiency. Microwave sintering [5,6] has a limited depth and requires a closed space, with strict requirements on the experimental environment during the sintering process. Local sintering consists of laser sintering [7,8,9,10], in which a laser spot scans the conductive pattern. This method has low efficiency and requires high local temperatures that may easily damage the substrate [11,12]. Sintering at room temperature is achieved by preparing a self-sintering conductive ink. Flash sintering [13,14,15,16,17,18,19,20] uses high-power pulsed xenon lamps to release energy for sintering conductive patterns in 1 to 2 ms. This technique benefits from having a large area of effect, being highly efficient, and doing little damage to the substrate.

However, in current flash sintering studies, energies as high as 1000–3000 J are released over a short time, the sintering effect is difficult to control, and the electrical conductivity of the conductive pattern after sintering is unstable and prone to excessive or incomplete sintering. Park et al. [13] and Sarkar et al. [14] reported that excessive energies cause significant sintering of the conductive pattern, which reduces its conductivity. Using a microscope, Joo et al. [15] and Yu et al. [18] observed that after experiencing inappropriate flash sintering, the surface of the conductive pattern was damaged and electrical conductivity decreased. Therefore, it is important to establish a sintering parameter prediction system to optimize sintering parameters for manufacturing. Yang et al. [21] reported changes in the sintered neck and atomic motion of nanoparticles of different sizes during sintering. Song and Wen [22] reported the molecular dynamics of different nano-nickel sizes during sintering, as well as shrinkage, gyration radius, mean square displacement, diffusivity, and activation energy results. Rahmani et al. [23] reported on the density and mechanical properties of nano-copper after sintering. Bansal and MacNeill et al. [24,25,26] modelled the variation of the temperature field and density during flash sintering of multi-metal particles. However, for inkjet printing and sintering of conductive patterns, sintering parameters and the conductivity of the corresponding conductive patterns are not linked in any simulation model.

The aim of this study was to solve a problem in existing sintering models—namely that they cannot predict the effects resulting from different sintering parameters. Herein, the heat balance equation is used to establish the temperature field of the conductive pattern during sintering; the temperature of the conductive pattern was observed to change over time under different flash power densities. Molecular dynamics methods were used to detail the sintering process of nano-silver particles: the viscosity of nanoclusters of different diameters at different temperatures was measured and the relationship between density and conductivity was derived by analysing the potential distribution and electronic density of different atomic spacings. Applying this result to multi-particle melting and sintering models based on the finite element method of fluid mechanics, conductivity under different sintering conditions was calculated for various sintered necks and volume fractions. Furthermore, the sintering state was predicted from sintering parameters. The state of conductive patterns with different sintering degrees was simulated, producing results that are consistent with the conductive patterns for different sintering degrees observed under an electron microscope. Simulations also reveal the reason why the conductive pattern is destroyed in the sintered state. Model results are expected to help optimize photon sintering technology and improve sintering accuracy.

## 2. Materials and Methods

**Figure 1:**
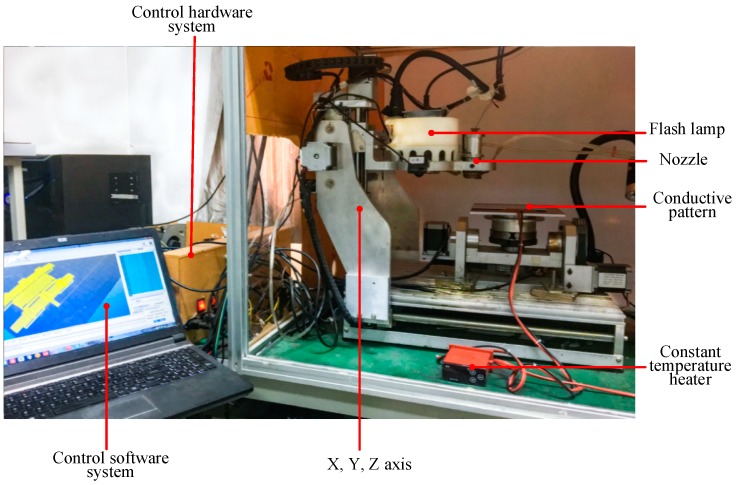
Sintering mechanism of silver nano-ink conductive pattern under flash irradiation.

As shown in Figure 1, the control software generates a print code, sends it to the control hardware, drives the three-axis motion to drive the piezoelectric nozzle for printing the conductive pattern, and sinters the conductive pattern using the pulsed xenon lamp.

The nano-silver conductive ink (Jet-100) is provided by HS Electronics Inc. (Suzhou, China), and the solvent is composed of an organic resin, ethanol, and the like. The piezoelectric nozzle (Micro-Fab Technologies Inc., Plano, TX, USA) has a diameter of 80 μm and a maximum injection frequency of 15 kHz. Flash sintering energy is provided by a 4 kW pulsed xenon lamp with a wavelength range of 230–680 nm and a sintering time of 1–1.5 ms. In these experiments, the sintering power density was adjusted to a range of 10–25 J/cm^2^, and sintering was conducted 1–5 times.

## 3. Modelling the Sintering of Nano-Silver Particles

**Figure 2:**
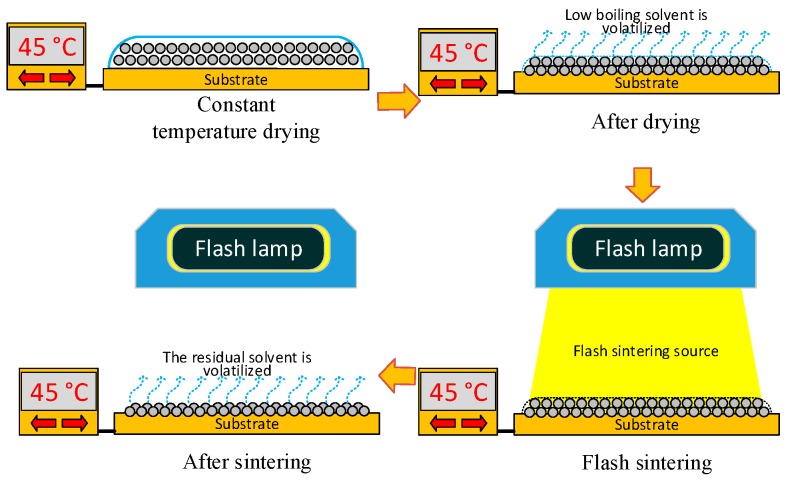
Printing-based sintering system.

**Figure 3:**
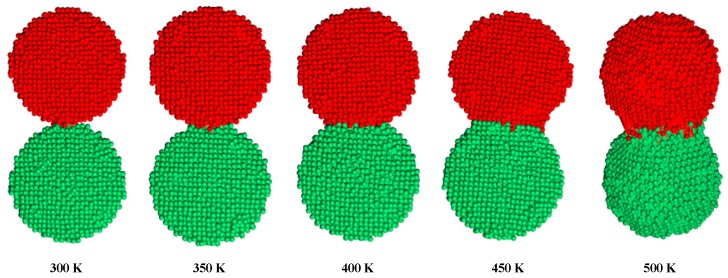
Molecular dynamics calculation of the sintered neck state of two nano-silver particles at different temperatures.

**Figure 4:**
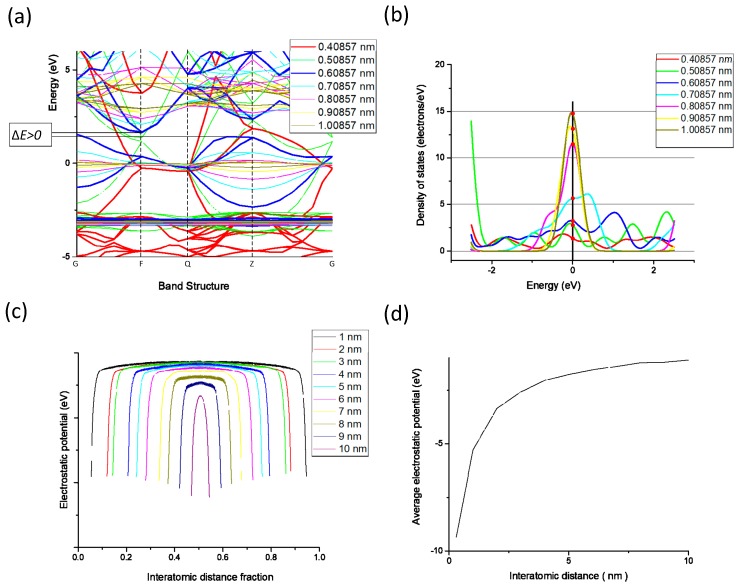
Comparison of (**a**) energy band structures of silver cells with different lattice constants; (**b**) density of states of silver cells with different lattice constants; and (**c**) potential distribution of silver atoms with different spacings; (**d**) average potential of silver atoms at different spacings.

As shown in Figure 2, the conductive pattern must be dried before sintering, and most of the solvent in the ink is volatilized. The temperature of the ink during the drying process is 40–50 °C, and the low-boiling-point solvent first volatilizes to form a flat semi-dry film. The flash lamp is then turned on for sintering, and while the middle- and high-boiling-point solvent is volatilized, the nano-silver particles connect to each other through the sintered neck to form a silver film, and the whole sample becomes electrically conductive. The characteristics of the nano-silver ink, flash sintering power density, and number of sintering processes all influence the conductivity of the pattern after sintering. Flash sintering releases 1000–3000 J of energy in 1–2 ms, making its effect difficult to control. This study used the molecular dynamics method to construct a nano-silver particle model with a diameter of 5 nm. The interaction between silver atoms uses embedded atomic potential. The internal atomic arrangement of the simulated silver clusters is created based on a face-centred cubic structure. The two nanoparticles were placed at a temperature of 298 K before sintering at a particle-to-particle distance of 0.5 nm. The heating rate was adjusted to 0.5 K/s, and the silver particles were sintered at a temperature of 300 K to 500 K. Figure 3 shows the different states achieved by adjusting the ambient temperature during sintering. As the temperature increases, a sintered silver neck begins to form between the nano-silver particles. The higher the temperature, the larger the size of the sintered neck, which in turn determines the sintered density and electrical conductivity of the pattern after sintering. Silver atomic cells with different lattice constants can be used to simulate the conductive pattern state of different sintered densities. The conductivity of the unit cell is derived by analysing the energy band structure and density of states of the silver atomic unit cell, as shown in Figure 4. Figure 4a shows that when the lattice constant is the parameter of the standard silver unit cell, there can be no grain boundary gap in the band structure. When the lattice constant reaches 0.60857 nm, a grain boundary gap begins to appear. As the lattice constant continues to increase, the boundary gap increases in size and the overall unit cell performance tends toward that of a semiconductor material. Figure 4b shows that the density of states on the Fermi surface increases with the lattice constant. The larger the density, the greater the electron bonding energy, the less likely it is to transition, and the poorer the conductivity of the material. Figure 4c shows the potential distribution of atomic spacings at the same field strength. The electrons are negatively charged and the potential near the atom drops because of the electron cloud around the silver atoms. The average of the different pitches is calculated in Figure 4d, and as the atomic spacing increases, the absolute value of the potential decreases. According to Ohm’s law, the potential is proportional to the current density and conductivity.

### 3.1. Fluid Dynamics Finite Element Model of Flash-Sintered Nanoparticles

According to the conductivity equation (Equation (1)), derived from Boltzmann’s equation [27], the electron concentration is proportional to the conductivity. From the nanocluster level, the atomic density is directly proportional to the electron density, so the conductivity can be determined by calculating the atomic density. Calculating many atomic models using molecular dynamics is time-consuming and inefficient, however. Using the fluid dynamics finite element method, it is possible to simulate the fusion of nano-silver particles with the surrounding nano-silver particles to form a sintered neck, obtain electrical conductivity, increase efficiency, and save time.

(1) $$\sigma =\frac{ne^{2}\tau }{m^{*}}$$

where *n* is the electron concentration, *τ* is the relaxation time, m* is the effective mass at the bottom of the band, and *e* is the electronic charge.

#### 3.1.1. Parameter Calculation

**Figure 5:**
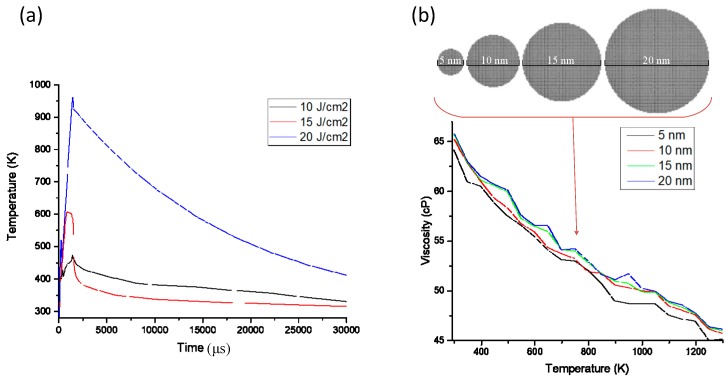
(**a**) Temperature vs. time under different flash sintering power densities; (**b**) viscosity of nano-silver particles of different diameters and temperatures.

The essence of flash sintering is to sinter the conductive pattern using thermal energy; however, the difference from conventional heat-based sintering is that the substrate damage is small after releasing a large amount of energy in 1–2 ms. When modelling, it is necessary to simplify the sintering process to affect temperature change by sintering power and frequency, which in turn affects the formation of the sintered neck between the nano-silver particles and is ultimately used to predict the conductive effect. The temperature field of the flash sintering process was analysed using commercial software (Comsol 5.3, COMSOL Inc., Stockholm, Sweden). A thermal model is established using the heat transfer equation, the flash sintering power density is set, and the temperature of the conductive pattern is determined by the numerical solution method using the heat transfer formula (Equation (2)). The model in Figure 5a shows the relationship obtained between time and temperature at different power densities. Viscosity is the most important parameter in the fluid calculation. The shear force can be calculated by the molecular dynamics method, and the dynamic viscosity can be derived for nano-silver particles of different sizes at different temperatures (Figure 5b).

(2) $$\rho \cdot C_{p}\cdot \frac{\partial T}{\partial t}+\nabla q=Q$$

where *ρ* is the density, *C_p_* is the constant-pressure specific heat capacity, *q* is the conducted heat flux, and *Q* is the energy source.

#### 3.1.2. Sintering Process

Comsol was used to establish a metal particle sintering model. The model assumes that the size of the nanoparticles is uniform and that the efficiency of accepting energy by the conductive pattern is constant at different time periods. Simulating the sintering process in different states requires the addition of the obtained temperature and viscosity parameters. The model simulates the mass and momentum transfer of fluids based on the Navier–Stokes equations of incompressible fluids. Surface tension is included to account for its effects:

(3) $$\rho \frac{\partial u}{\partial t}+\rho (u\cdot \nabla )u=\nabla \cdot [-pI+\mu (\nabla u+{\left(\nabla u\right)}^{T})]+F_{\mathrm{st}}+\rho g$$

where *ρ* is the density, *μ* is the dynamic viscosity, *p* is the pressure, g is the gravity vector, and *F*_st_ is the surface tension acting between the air and particle interfaces. When ∇·u=0, the equation represents an incompressible fluid.

(4) $$F_{\mathrm{st}}=\nabla \cdot (\sigma (Ι-(nn^{T}))\delta )$$

where I is the identity matrix, *n* is the interface normal, *σ* is equal to the surface tension coefficient (N/m), and *δ* is the Dirac function.

**Figure 6:**
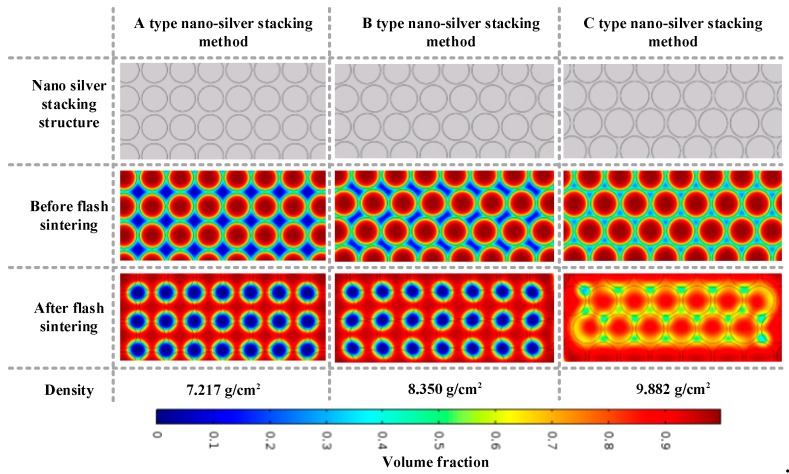
Model of multi-particle sintering at different densities.

A multi-particle model with a nano-silver particle diameter of 5 nm was constructed to simulate the state change of the conductive pattern during sintering. The sintering model of different sintered necks is obtained by different nano-silver particle alignment methods, as shown in Figure 6. The temperature and viscosity data were brought into the model for numerical solutions to obtain the volume fraction distribution, and the volume fraction was weighted to obtain the density of the conductive pattern under different alignment methods and calculate the conductivity. Figure 6 illustrates that the nano-silver particles are independent before sintering, and when the sintering is complete, a stable sintered neck is formed between the particles. In addition, the sintering gap and density differ with different arrangement modes. Furthermore, the minimum density of method A is 7.217 g/cm^3^, whereas the density of method B is, at most, 9.882 g/cm^3^.

## 4. Results and Discussion

**Figure 7:**
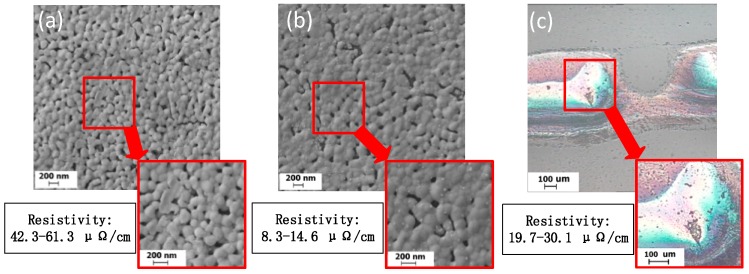
Field-effect scanning electron microscopy images of (**a**) incompletely sintered silver film; (**b**) optimally sintered silver film; and (**c**) the damaged surface of an over-sintered silver film.

**Figure 8:**
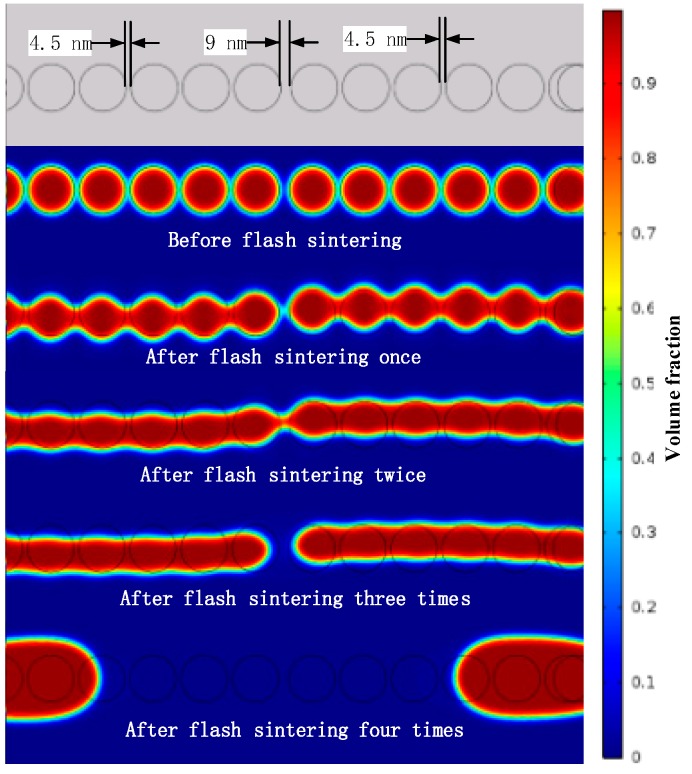
The sintering simulation model reveals the principle of excessive sintering leading to surface damage.

In existing references [3,4,14,15,16,17,18,19], the nano-silver conductive pattern resistivity can be as low as 7 μΩ/cm to 10 μΩ/cm. The conductive patterns of different sintering degrees were observed using a scanning electron microscope, as shown in Figure 7. When sintering is incomplete, as shown in Figure 7a, the nano-silver particles are independent, no clear sintered neck is formed, and the electrical resistivity is high (42.3–61.3 μΩ/cm). When the sintering process is complete, however, as shown in Figure 7b, a distinct sintered neck is formed between the nano-silver particles and the electrical resistivity is in a reasonable range (8.3–14.6 μΩ/cm). When over-sintering occurs, as shown in Figure 7c, the surface of the conductive pattern is significantly damaged, the nano-silver particles converge remarkably, and the electrical resistivity of the overall conductive pattern is increased. In this state, the nano-silver particles are not uniformly distributed, as shown in Figure 8. When sintering is performed twice, there are still sintered necks between the connected nano-silver particles. In the actual sample, the nanoparticles are randomly arranged on the substrate and nanoparticle spacing cannot be ensured to be the same, which alters the state of the sintered neck formed by the same nanoparticle and the surrounding particles. When sintering continues, the excessive sintering energy drives the nano-silver particles to decrease in viscosity and possess liquid characteristics. The whole sample begins to flow from low to high surface tension and the force differs because of the difference in the sintered necks on both sides, resulting in single-side sintering. The neck is torn open, eventually causing nano-silver particle aggregation.

**Figure 9:**
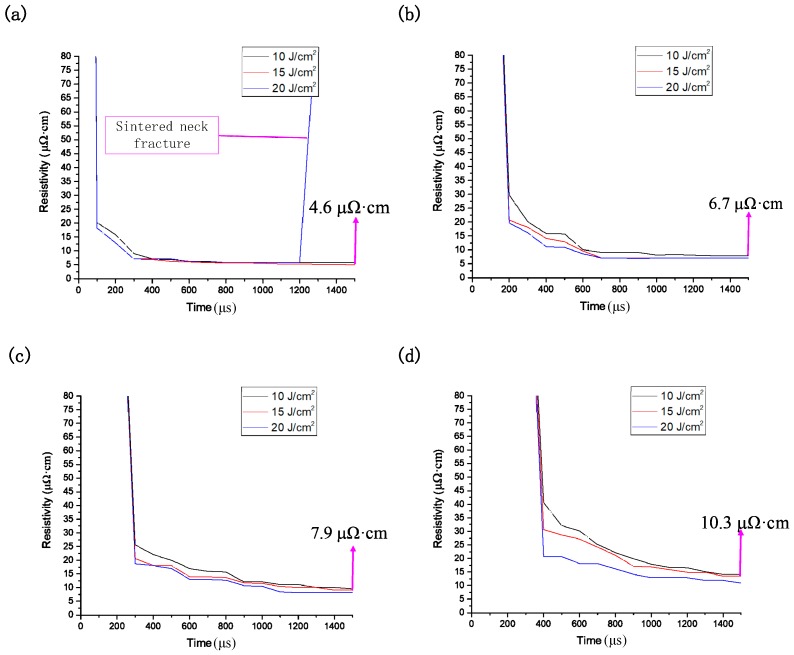
Simulation of the change in resistivity of different sintered power densities for nanoparticle radii of (**a**) 5 nm; (**b**) 10 nm; (**c**) 15 nm; and (**d**) 20 nm.

The conductivity can be derived from the size of the sintered necks. The resistivity of pure silver is known to be 1.65 μΩ/cm. Different sintered neck sizes lead to different conductive pattern densities; the density of conductive patterns is proportional to the electrical conductivity by electrical property analysis of different lattice constant silver clusters, which is inversely proportional to the resistivity. According to this rule, the change in resistivity produced by different sintering power densities applied to different nano-silver diameters can be derived. As shown in Figure 9, resistivity decreases with power, but resistance is still observed in Figure 9a. The rate suddenly increases at a sintering of 20 J/cm^2^ because the sintering power is too high, as shown in Figure 7c. In addition, the nano-silver particles melt into each other and destroy the surface of the conductive pattern. As the diameter of the nano-silver increases, the lowest resistivity that can be obtained under the same sintering conditions also increases because the larger the diameter of the nano-silver particles, the higher the sintering energy required to drive the sintering process. By integrating the data, a set of prediction models can be established. According to the sintering parameters and the parameters of the nano-silver particles in the conductive ink, the conductivity of the sintered conductive pattern can be predicted, which can significantly reduce the experiment duration and effectively reduce the production costs of the testing or manufacturing processes.

## 5. Conclusions

This study provided an in-depth analysis of the state change in nano-silver conductive patterns during sintering. By analysing the band structure and density of silver crystals with different lattice constants, the relationship between the density of the silver atom and conductivity was derived. As per the Boltzmann equation, electron density is proportional to conductivity and atomic density. A fluid dynamics finite element method was used to simulate multiple nano-silver particles and the change in dynamic viscosity of nano-silver particles at different temperatures. The temperature change data at different sintering powers were also added to the model. The density of the conductive pattern was obtained according to the size of the formed sintered neck to predict the conductivity of the conductive pattern. The state of nano-silver particles with different sintering degrees, obtained by scanning electron microscopy, agreed with the model results and revealed the mechanism responsible for the destruction of over-sintered surfaces in the conductive pattern. The model results in this paper are expected to help optimize photon sintering technology and promote technological progress.

## References

[1] Neil C., Hutchings I.M., Martin G.D. (2012). Printed circuit board fabrication. Inkjet Technology for Digital Fabrication.

[2] Kamyshny A., Steinke J., Magdassi S. (2011). Metal-based inkjet inks for printed electronics. Open Appl. Phys. J..

[3] Niittynen J., Abbel R., Mäntysalo M., Perelaer J., Schubert U.S., Lupo D. (2014). Alternative sintering methods compared to conventional thermal sintering for inkjet printed silver nanoparticle ink. Thin Solid Film.

[4] Greer J.R., Street R.A. (2007). Thermal cure effects on electrical performance of nanoparticle silver inks. Acta Mater..

[5] Perelaer J., de Gans B.-J., Schubert U.S. (2006). Ink-jet printing and microwave sintering of conductive silver tracks. Adv. Mater..

[6] Perelaer J., Klokkenburg M., Hendriks C.E., Schubert U.S. (2009). Microwave flash sintering of inkjet-printed silver tracks on polymer substrates. Adv. Mater..

[7] Chiolerio A., Maccioni G., Martino P., Cotto M., Pandolfi P., Rivolo P., Ferrero S., Scaltrito L. (2011). Inkjet printing and low power laser annealing of silver nanoparticle traces for the realization of low resistivity lines for flexible electronics. Microelectron. Eng..

[8] Hong S., Yeo J., Kim G., Kim D. (2007). Nonvacuum, Maskless Fabrication of a Flexible Metal Grid Transparent Conductor by Low-Temperature Selective Laser Sintering of Nanoparticle Ink. Acs Nano.

[9] Agarwala S., Goh G.L., Dinh Le T.-S., An J., Peh Z.K., Yeong W.Y., Kim Y.-J. (2018). Wearable Bandage based Strain Sensor for Home Healthcare: Combining 3D Aerosol Jet Printing and Laser Sintering. Acs Sens..

[10] Ko S.H., Pan H. (2007). All-inkjet-printed flexible electronics fabrication on a polymer substrate by low-temperature high-resolution selective laser sintering of metal nanoparticles. Nanotechnology.

[11] Magdassi S., Grouchko M., Berezin O., Kamyshny A. (2010). Triggering the Sintering of Silver Nanoparticles at Room Temperature. Acs Nano.

[12] Grouchk M., Kamyshny A. (2011). Conductive Inks with a “Built-In” Mechanism That Enables Sintering at Room Temperature. Acs Nano.

[13] Park S.-H., Kim H.-S. (2014). Flash light sintering of nickel nanoparticles for printed electronics. Thin Solid Film.

[14] Sarkar S.K., Gupta H., Gupta D. (2017). Flash light sintering of silver nanoink for inkjet-printed thin-film transistor on flexible substrate. IEEE Trans. Nanotechnol..

[15] Joo S.-J., Hwang H.-J., Kim H.-S. (2014). Highly conductive copper nano/microparticles ink via flash light sintering forprinted electronics. Nanotechnology.

[16] Perelaer J., Abbel R., Wünscher S., Jani R., van Lammeren T.U.S. (2012). Schubert, Roll-to-roll compatible sintering of inkjet printed features by photonic and microwave exposure: From non-conductive ink to 40% bulk silver conductivity in less than 15 seconds. Adv. Mater..

[17] Kang J.S., Ryu J., Kim H.S., Hahn H.T. (2011). Sintering of inkjet-printed silver nanoparticles at room temperature using intense pulsed light. J. Electron. Mater..

[18] Yu M.-H., Joo S.-J., Kim H.-S. (2017). Multi-pulse flash light sintering of bimodal Cu nanoparticle-ink for highly conductive printed Cu electrodes. Nanotechnology.

[19] Akhavan V., Schroder K., Farnsworth S. (2017). Photonic Curing Enabling High-Speed Sintering of Metal Inkjet Inks on Temperature-Sensitive Substrates. Handb. Ind. Inkjet Print. Full Syst. Approach.

[20] Albrecht A. (2016). Inkjet printing and photonic sintering of silver and copper oxide nanoparticles for ultra-low-cost conductive patterns. J. Mater. Chem. C.

[21] Yang L., Gan Y., Zhang Y., Chen J.K. (2012). Molecular dynamics simulation of neck growth in laser sintering of different-sized gold nanoparticles under different heating rates. Appl. Phys. A.

[22] Song P., Wen D. (2010). Molecular dynamics simulation of the sintering of metallic nanoparticles. J. Nanopart. Res..

[23] Rahmani F., Jeon J., Jiang S., Nouranian S. (2018). Melting and solidification behavior of Cu/Al and Ti/Al bimetallic core/shell nanoparticles during additive manufacturing by molecular dynamics simulation. J. Nanopart. Res..

[24] Dexter M., Bhandari R., Chang C.-H. (2017). Controlling processing temperatures and self-limiting behaviour in intense pulsed sintering by tailoring nanomaterial shape distribution. Rsc Adv..

[25] Bansal S., Malhotra R. (2016). Nanoscale-shape-mediated coupling between temperature and densification in intense pulsed light sintering. Nanotechnology.

[26] MacNeill W., Choi C.H., Chang C.H., Malhotra R. (2015). On the self-damping nature of densification in photonic sintering of nanoparticles. Sci. Rep..

[27] Kun H. (1966). Solid State Physics.

